# Effects of drought stress on photosynthesis and photosynthetic electron transport chain in young apple tree leaves

**DOI:** 10.1242/bio.035279

**Published:** 2018-11-15

**Authors:** Zhibo Wang, Guofang Li, Hanqing Sun, Li Ma, Yanping Guo, Zhengyang Zhao, Hua Gao, Lixin Mei

**Affiliations:** 1 Key Laboratory of Horticulture Plant Biology and Germplasm Innovation in Northwest China, Yangling, Shaanxi 712100, China; 2 College of Horticulture, Northwest A&F University, Yangling, Shaanxi 712100, China

**Keywords:** Photosynthetic electron transport chain, Antioxidant enzymes, D1 protein, Reactive oxygen species, Water stress

## Abstract

In our study, the effects of water stress on photosynthesis and photosynthetic electron transport chain (PETC) were studied in several ways, including monitoring the change of gas exchange parameters, modulated chlorophyll fluorescence, rapid fluorescence induction kinetics, reactive oxygen species (ROS), antioxidant enzyme activities and D1 protein levels in apple leaves. Our results show that when leaf water potential (*ψ*_w_) is above –1.5 MPa, the stomatal limitation should be the main reason for a drop of photosynthesis. In this period, photosynthetic rate (*P*_N_), stomatal conductance (*G*_s_), transpiration rate (*E*) and intercellular CO_2_ concentration (*C*_i_) all showed a strong positive correlation with *ψ*_w_. Modulated chlorophyll fluorescence parameters related to photosynthetic biochemistry activity including maximum photochemical efficiency (F_v_/F_m_), actual photochemical efficiency of PSII (Φ_PSII_), photochemical quenching coefficient (*q*_P_) and coefficient of photochemical fluorescence quenching assuming interconnected PSII antennae (*q*_L_) also showed a strong positive correlation as *ψ*_w_ gradually decreased. On the other hand, in this period, Stern-Volmer type non-photochemical quenching coefficient (NPQ) and quantum yield of light-induced non-photochemical fluorescence quenching [*Y*_(NPQ)_] kept going up, which shows an attempt to dissipate excess energy to avoid damage to plants. When *ψ*_w_ was below –1.5 MPa, *P*_N_ continued to decrease linearly, while *C*_i_ increased and a ‘V’ model presents the correlation between *C*_i_ and *ψ*_w_ by polynomial regression. This implies that, in this period, the drop in photosynthesis activity might be caused by non-stomatal limitation. F_v_/F_m_, Φ_PSII_, *q*_P_ and *q*_L_ in apple leaves treated with water stress were much lower than in control, while NPQ and *Y*_(NPQ)_ started to go down. This demonstrates that excess energy might exceed the tolerance ability of apple leaves. Consistent with changes of these parameters, excess energy led to an increase in the production of ROS including H_2_O_2_ and O_2_^•^−^^. Although the activities of antioxidant enzymes like catalase (CAT), superoxide dismutase (SOD) and peroxidase (POD) increased dramatically and ascorbate peroxidase (APX) decreased in apple leaves with drought stress, it was still not sufficient to scavenge ROS. Consequently, the accumulation of ROS triggered a reduction of net D1 protein content, a core protein in the PSII reaction center. As D1 is responsible for the photosynthetic electron transport from plastoquinone A (Q_A_) to plastoquinone B (Q_B_), the capacity of PETC between Q_A_ and Q_B_ was considerably downregulated. The decline of photosynthesis and activity of PETC may result in the shortage of adenosine triphosphate (ATP) and limitation the regeneration of RuBP (*J*_max_), a key enzyme in CO_2_ assimilation. These are all non-stomatal factors and together contributed to decreased CO_2_ assimilation under severe water stress.

## Abstract (precis)

**Summary:** This research reports that drought-induced photosynthesis declines due to stomatal and non-stomatal limitation, which was PSII photoinhibition-dependent on D1 protein and over-reduces the electron transport chain.

## INTRODUCTION

Water availability is an important factor affecting plant growth and yield in arid and semi-arid regions, where plants are often subjected to periods of drought (Chaves et al., 2003). Under drought stress conditions, many metabolic processes, including photosynthesis, are negatively affected. For instance, water deficiency damages basic organization structure, which inhibits carbon assimilation and damages photosynthetic apparatus (Ali and Ashraf, 2011; Golldack et al., 2011). Previous studies have illustrated the decrease in photosynthesis of leaves is usually caused by stomatal limitation under mild to moderate drought conditions and non-stomatal limitation under severe drought conditions (Degl'Innocenti et al., 2009; Misson et al., 2010).

Such a decrease in photosynthesis leads to plants absorbing more light energy than can be consumed by photosynthetic carbon fixation. This excess energy has the potential to trigger an increase in the production of reactive oxygen species (ROS) including O_2_^•^−^^ and H_2_O_2_, which has been proven to hinder the synthesis of PSII core D1 (Murata and Takahashi, 2008). Consistent with the inhibition of D1 synthesis, the activity of photosynthetic electron transport chain (PETC) also downregulates.

Furthermore, some previous studies indicated the fixation of CO_2_ in the Calvin cycle is sensitive to environmental stresses including high-temperature stress, low-temperature stress (Greer et al., 1986) and salt stress (Altaweel et al., 2007). Under these environmental stresses, the inhibition of the synthesis of D1 protein due to interruption of the fixation of CO_2_ might be expected to accelerate the decrease in photosynthesis. Nevertheless, it remains unclear (1) how the drought stress impacts the turnover of D1 protein and activity of PETC and (2) how the photosynthesis and PETC interact especially in the non-stomatal limiting phase under drought stress conditions.

In the present study, leaf water potential (*ψ*_w_) and gas exchange parameters including net photosynthetic rate (*P*_N_), intercellular CO_2_ concentration (*C*_i_), transpiration rate (*E*) and stomatal conductance (*G*_s_) are utilized to explore the main reason for the decrease in photosynthesis of apple leaves under different drought stress levels. Through analysis of chlorophyll *a* fluorescence and the determination of D1 protein content, we can assess the activity of photosynthetic apparatus, including PETC. The content of O_2_^•^−^^ and H_2_O_2_ and antioxidant enzymes activities were also used for probing the damage level to photosynthesis of apple leaves brought from water deficiency. All of the above techniques were applied to this study in order to investigate how water stress impacts the turnover of D1 protein, activity of PETC and the relationship between photosynthesis and PETC, especially in the non-stomatal limiting phase under water stress conditions.

## RESULTS

### *ψ*_w_ and gas exchange

**Fig. 1.**
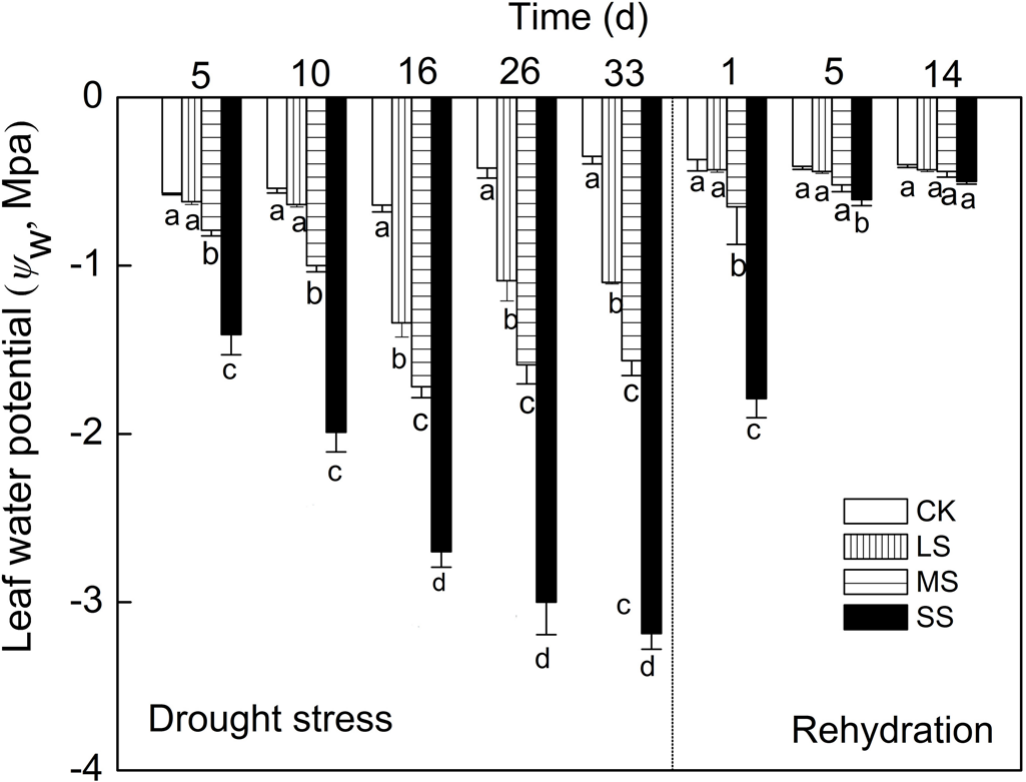
**Responses of *ψ*_w_ to water stress and subsequent rehydration in apple leaves.** Different letters indicate significant difference by Tukey tests at *P*<0.05. CK, control group. Values are means±s.e. (*n*=6).

*ψ*_w_ was sensitive to drought conditions and affected by different intensities of drought stress and subsequent rehydration. The *ψ*_w_ of control plants was higher than those of plants subjected to slight stress (LS), moderate stress (MS) or severe stress (SS). On day 5 and 10, the *ψ*_w_ of LS plants was approximately equal to control and decreased significantly after 16 days. The *ψ*_w_ decreased significantly in MS and SS plants throughout the stress period. The *ψ*_w_ of SS plants dropped to −3.19 MPa on day 33 (Fig. 1), in which leaves wilted seriously and some leaf margin dried up. After rehydration, water status of all stressed plants recovered to control level and plants with different stress treatments showed different recovery rates; specifically, the *ψ*_w_ of LS plants recovered within 1 day while MS and SS plants took over 5 days.

**Fig. 2.**
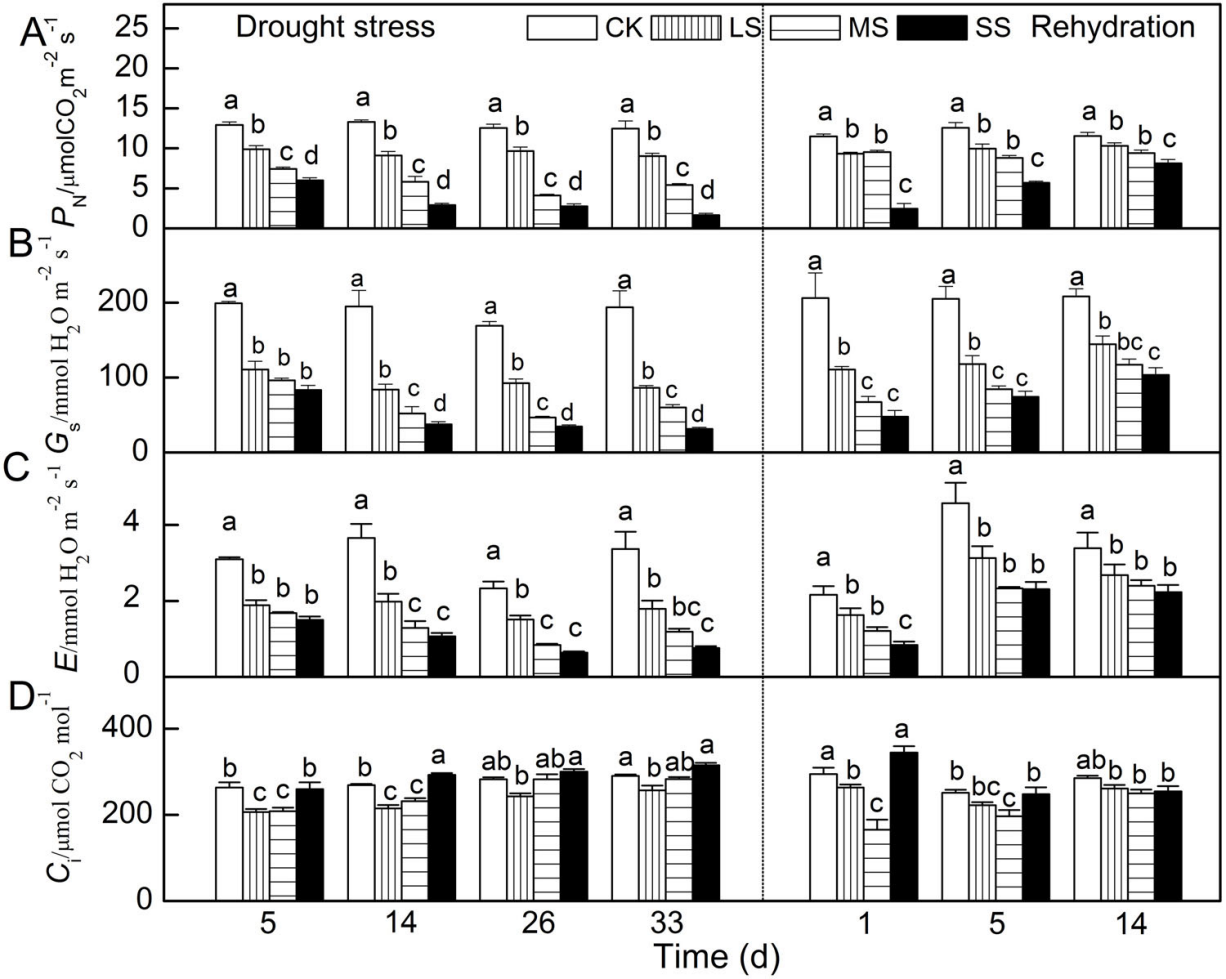
**Responses of gas exchange parameters to water stress and subsequent rehydration in apple leaves.** (A) *P*_N_. (B) *G*_s_. (C) *E*. (D) *C*_i_. Different letters indicate significant difference by Tukey tests at *P*<0.05. Values are means±s.e. (*n*=6).

*P*_N_, *G*_s_, *E* and *C*_i_ were also influenced differently by imposed drought stress and subsequent rehydration. In comparison with control, *P*_N_, *G*_s_ and *E* decreased gradually as stress proceeded (Fig. 2). After 33 days of drought stress treatments, when compared with control, *P*_N_, *G*_s_ and *E* of LS, MS and SS plants decreased 28%, 57% and 87% (LS); 56%, 69% and 84% (MS) and 47%, 65% and 78% (SS), respectively.

Unlike the three parameters above, *C*_i_ of LS and MS plants went down after 5 days of treatment and showed a trend of increasing over time. Meanwhile, *C*_i_ of SS plants remained at a high level and steady state after 14 days of treatment. After rehydration, *P*_N_, *G*_s_ and *E* of all stressed plants gradually increased and recovered to levels of control to different extents. Specifically, *P*_N_, *G*_s_ and *E* of LS, MS and SS plants recovered to 90%, 82% and 71% (LS); 69%, 56% and 50% (MS) and 79%, 71% and 66% (SS) of control group after 14 days, respectively. However, after rehydration, *C*_i_ of LS, MS and SS plants all fell first and grew later, unlike *P*_N_, *G*_s_ and *E*.

**Fig. 3.**
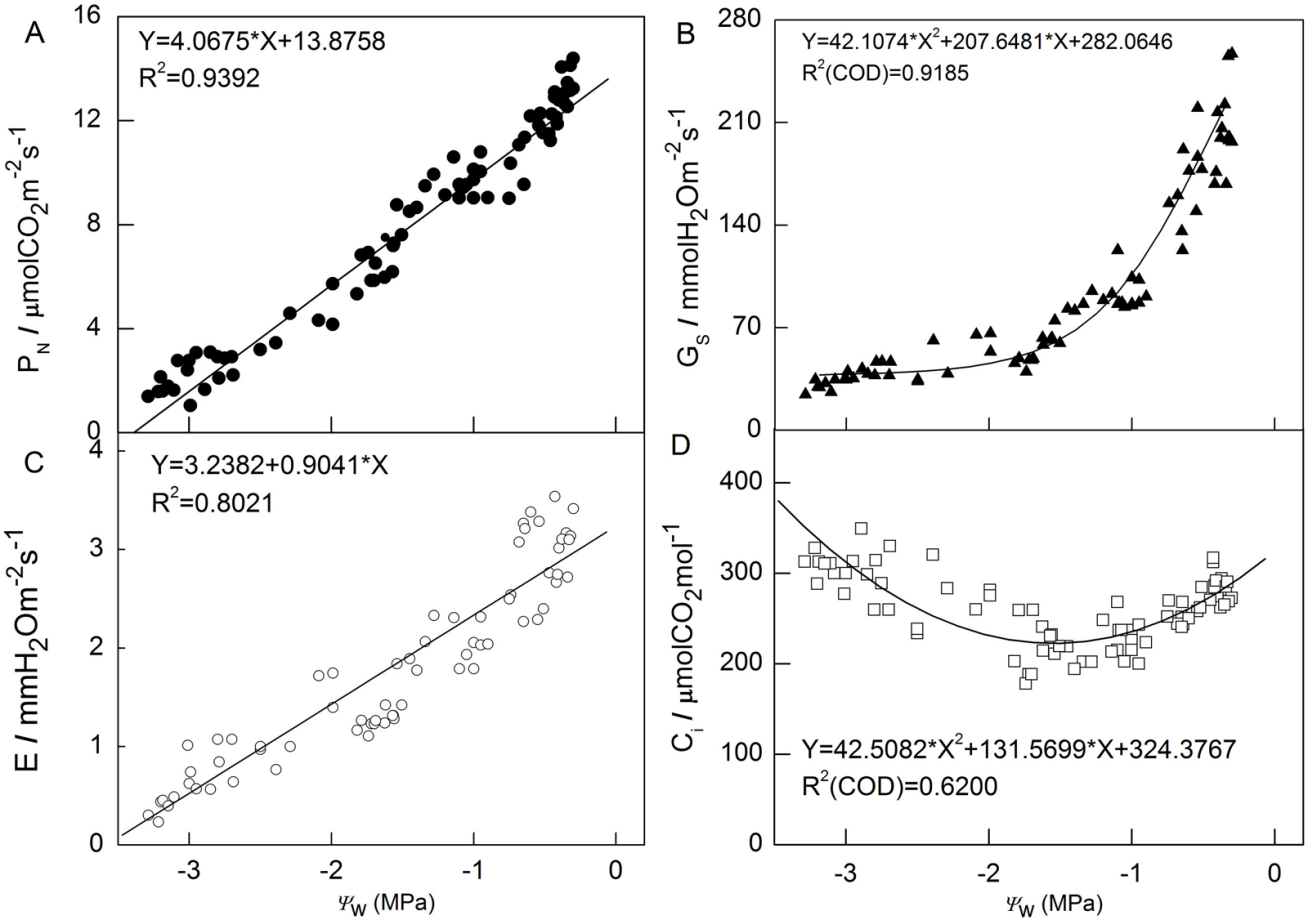
**Correlation analysis between gas exchange parameters and *ψ*_w_ of apple tree leaves under water stress.** (A) *P*_N_. (B) *G*_s_. (C) *E*. (D) *C*_i_. The coefficients of determination (R^2^) were calculated using the data from all treatments.

Furthermore, in order to analyze the relationship between drought stress and gas exchange parameters, we calculated correlation coefficients the between *ψ*_w_ and *P*_N_, *G*_s_, *E* and *C*_i_ (Fig. 3). A positive linear regression correlation between *P*_N_ and *ψ*_w_ was seen, with the coefficient reaching 0.9392. Similar correlation existed between *E* and *ψ*_w_ with a smaller coefficient 0.8021. The correlation coefficient of *G*_s_ between *ψ*_w_ was 0.9185 and that of *C*_i_ between *ψ*_w_ was 0.6200; their polynomial regression has the same turning point at approximately −1.40 MPa. In general, *P*_N_, *G*_s_ and *E* had a positive correlation with *ψ*_w_, while *C*_i_ had a ‘V’ model correlation.

Because of this, we investigated whether Rubisco carboxylation and RuBP regeneration might be limiting during drought stress by measuring the *P*_N_/*C*_i_ response, and calculated the value of both the maximum velocity of Rubisco for carboxylation (*V*_cmax_) and the maximum rate of electron transport (*J*_max_). On day 12, for control group, LS, MS and SS the *V*_cmax_ values were 76.55, 74.82, 51.96 and 23.24 µmol m^−2^ s^−1^, respectively; the *J*_max_ values were 80.71, 70.97, 58.34 and 41.24 µmol m^−2^ s^−1^, respectively. In MS and SS, drought stress reduced *V*_cmax_ and *J*_max_ significantly; these results suggest that MS and SS have a major impact on RuBP regeneration capacity and RuBP carboxylase activity, but LS has a lesser effect on RuBP carboxylase activity.

### Modulated chlorophyll fluorescence

During drought stress conditions, maximum photochemical efficiency (F_v_/F_m_) and F_v_′/F_m_′ both decreased after 33 days of treatment (Table 1). After 5 days of water deficit, F_v_′/F_m_′ of SS plants was lower than others. Actual photochemical efficiency of PSII (Φ_PSII_) had similar trends throughout the experiments; it decreased significantly with increased intensity of water stress on day 33. Φ_PSII_ of SS plants decreased to 24% of control group on day 33. Interestingly, similar trends existed in *q*_P_ and coefficient of photochemical fluorescence quenching assuming interconnected PSII antennae (*q*_L_). In addition, Stern-Volmer type non-photochemical quenching coefficient (NPQ) and Y_(NPQ)_ with drought treatments all increased on day day 5. But on day 33, Y_(NPQ)_ of SS plants decreased compared with MS plants, and at the same time, NPQ of SS plants dropped to minimum in all plants.

**Table 1.**
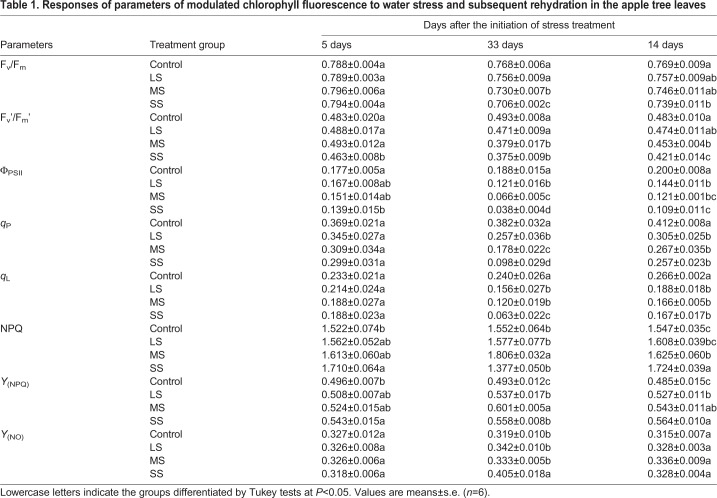
**Responses of parameters of modulated chlorophyll fluorescence to water stress and subsequent rehydration in the apple tree leaves**

It is noteworthy that, although 14 days of rehydration made *ψ*_w_ recover to pre-drought stress levels, it was not sufficient for total recovery in parameters of chlorophyll fluorescence, especially in MS and SS plants. After 14 days of rehydration, compared with control group, chlorophyll fluorescence parameters in stressed plants recovered in different degrees.

### Rapid fluorescence induction kinetics

**Fig. 4.**
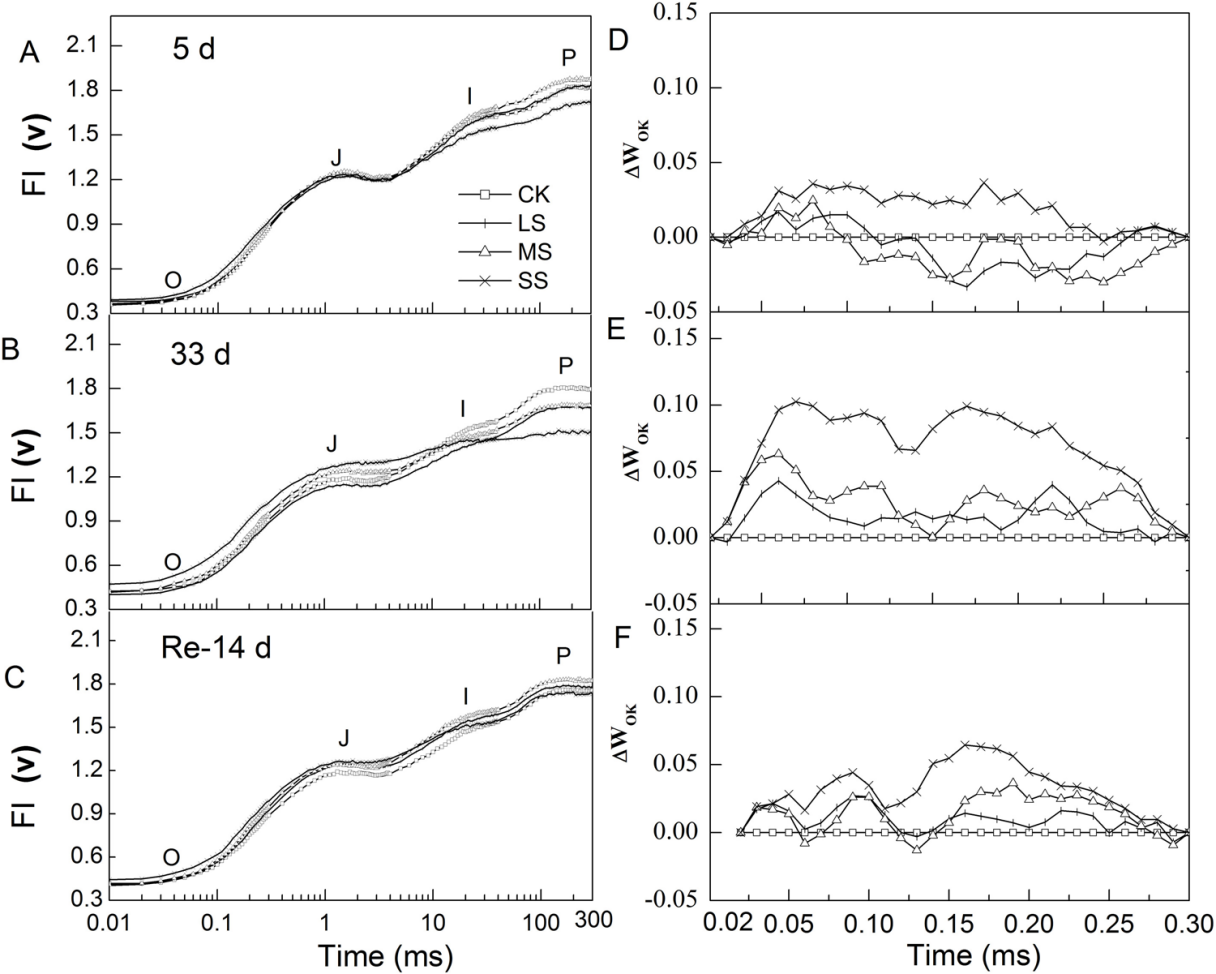
**Responses of chlorophyll *a* fluorescence transient (OJIP) and L-Band to different water stress treatments.** (A-F) Reactions for 5 days (A,D), 33 days (B,E) and then rehydration treatment for 14 days (C,F) in apple leaves. *V*_OK_=(*F*_t_−*F*_O_)/(*F*_300µs_−F_O_), Δ*V*_OK_=*V*_OK(treatment)_-*V*_OK(control)_. Values are means±s.e. (*n*=6).

All rapid fluorescence induction kinetics exhibited a typical polyphasic OJIP curve, where O was original fluorescence (initial fluorescence, F_0_), J and I sites were intermediate transients, and P was the peak (maximal fluorescence, F_m_) (Fig. 4). On the left column in Fig. 4, all transients had similar trends in Fig. 4A,C, while there were significant differences in Fig. 4B. F_0_ of SS plants significantly increased on day 33, while there was little difference between day 5 and after 14 days of rehydration. The fluorescence intensity (F_I_) of the J site in SS plants was significantly higher than those in control group and LS plants, while it was lower on the I site compared with control group and LS. On the right column in Fig. 4, OJIP curves with different treatments were normalized (L-band) between O and K (300 μs) sites. The value at about 150 μs in the L-band is an indicator of the energetic connectivity among PSII units and the high value means low connectivity. As shown in Fig. 4D, the L-band of SS plants had a positive value on day 5, while negative values were present in MS and LS plants. On day 33, all L-bands in the three stress treatments had positive values and the value was higher with increasing intensity of stress. After 14 days of rehydration, the L-bands of LS and MS plants recovered close to control group level, but that of SS plants was still remarkably higher than control group (Fig. 4F).

### Western blot analysis of D1 protein

**Fig. 5.**
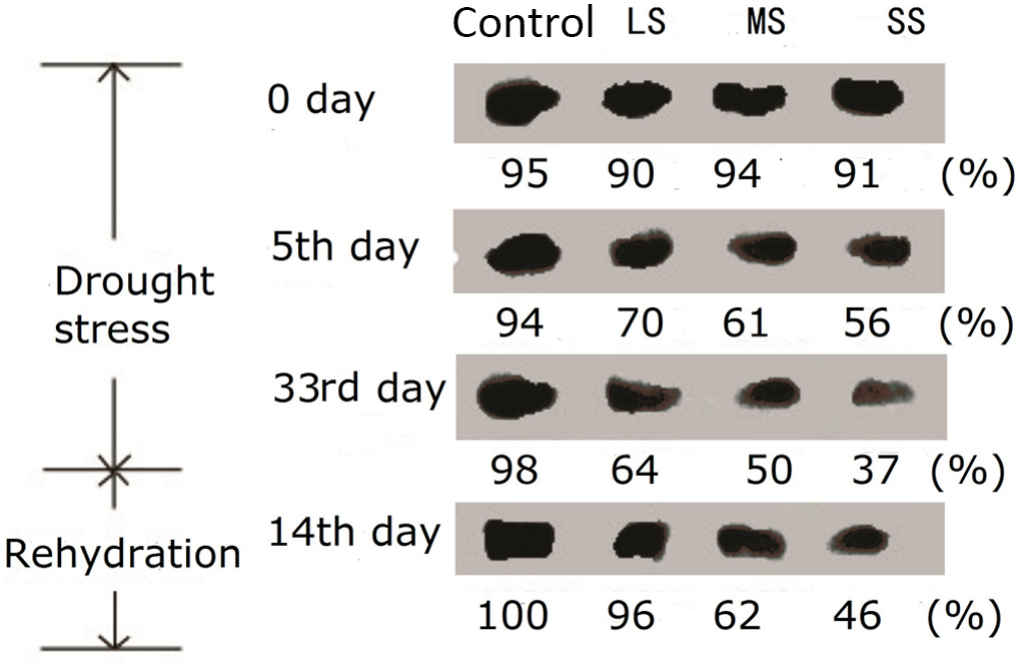
**D1 protein contents with different water stress treatments for 0 day, 5 days, 33 days and rehydration treatment for 14 days.** Quantitative analysis for the content of D1 protein is completed using gray analysis by Quantity One (Bio-Rad). And the content of D1 protein in the control with rehydration treatment for 14 days is chosen as the reference (100%).

To prove that the drought stress damaged key site of photosynthetic apparatus may be on the photosynthetic electron transport from Q_A_ to Q_B_, western blot analysis with an antibody against the D1 protein was conducted (Fig. 5). A significant reduction was observed, and with the increase of the stress intensity and extension of the treatment time, the difference in D1 contents between drought treated plants and control group went up. After 14 days of rehydration the photosynthetic operation was improved due to elevated D1 synthesis, but not enough to recover to control group level.

### Accumulation of ROS and change of antioxidant enzyme activities

Our results showed clearly that 33 days of drought stress induced a higher generation rate of O_2_^•^−^^ and greater H_2_O_2_ contents (Table 2). With the enhancement of drought stress intensity, the contents of O_2_^•^−^^ and H_2_O_2_ were significantly higher than in control group.

**Table 2.**
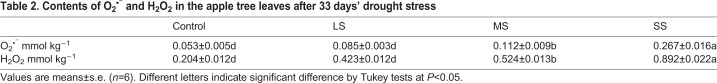
**Contents of O_2_^•^−^^ and H_2_O_2_ in the apple tree leaves after 33 days' drought stress**

**Fig. 6.**
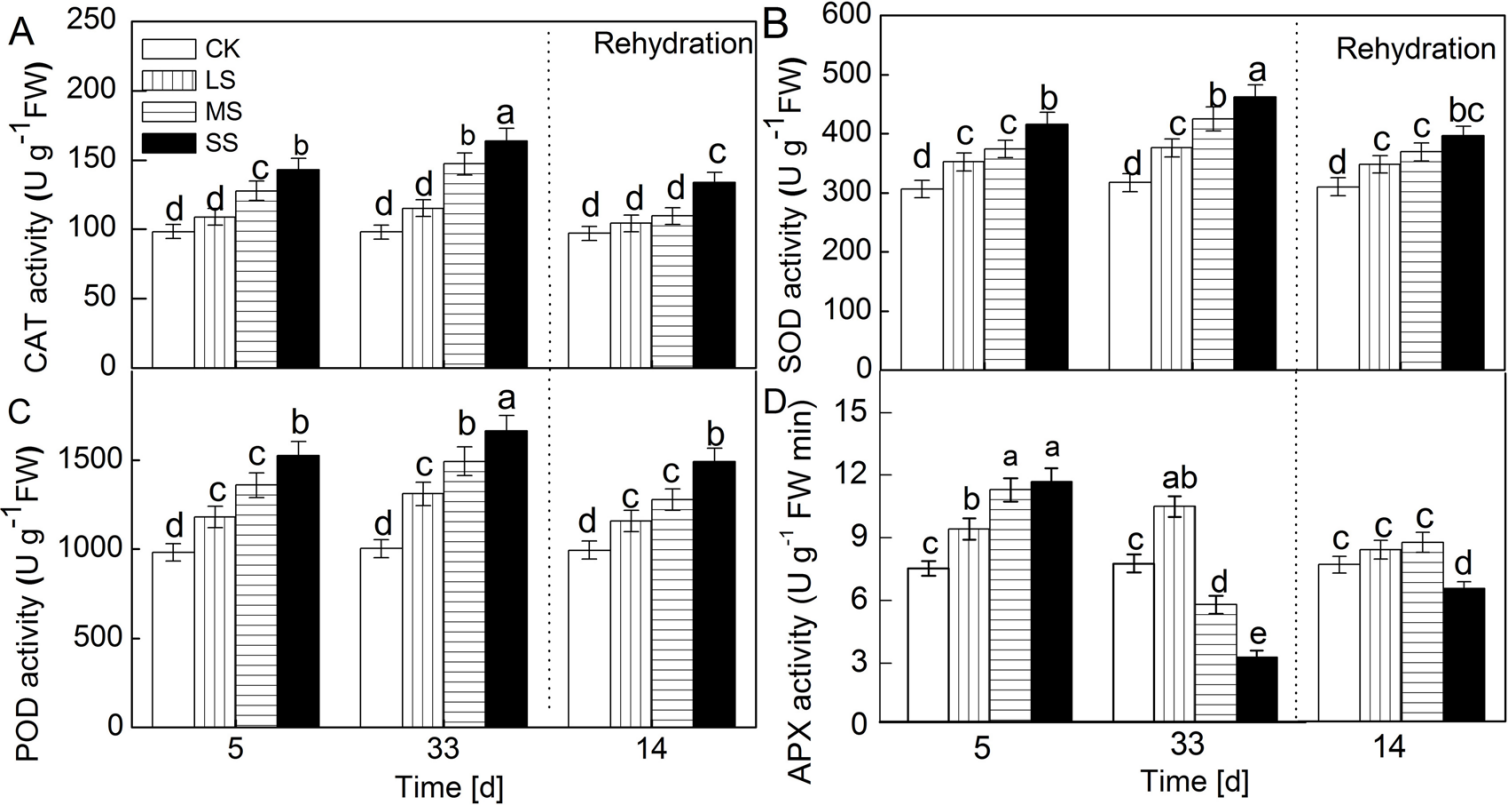
Change in the activity of CAT (A), SOD (B), POD (C) and APX (D) with different water stress treatments for 5 days, 33 days and rehydration treatment 14 days. Values are means±s.e. (*n*=6).

Significant increases were illustrated in the activities of antioxidant enzymes in drought-stressed plants (Fig. 6). Catalase (CAT) activity in the leaves put through drought stress treatments was much higher than that in control group. A similar response to drought was seen in the activities of superoxide dismutase (SOD) and peroxidase (POD). On the other hand, the change in ascorbate peroxidase (APX) activity was markedly different than those in antioxidant enzymes under MS and SS conditions; APX activity declined at day 33. After 14 days of rehydration, compared with control group, the activities of three antioxidant enzymes in stressed plants recovered by different degrees, but these parameters did not recover to control group level, especially in APX.

## DISCUSSION

*ψ*_w_ can be regarded as an indicator to effectively assess water status of plants (Lima et al., 2002). In the present study, *ψ*_w_ decreased with the degree and duration of drought stress treatments (Fig. 1). Gas exchange, which was *P*_N_, *G*_s_ and *E* decreased significantly and they were closely related to the degree and duration of drought stress (Fig. 2). These parameters were all found to have a strong relationship with *ψ*_w_ (Fig. 3). Besides the linear correlation between *P*_N_ and *ψ*_w_ (Fig. 3A; Šimpraga et al., 2011; Sun et al., 2013), a simple positive regression correlation was also found between *G*_s_ or *E* and *ψ*_w_ (Fig. 3B,C). At the earlier stage of drought stress, the plummet in *G*_s_ suggests that a reduction in stomatal conductance can have protective effects because it allows the plant to save water and to improve its efficient use (Chaves et al., 2009). As some studies indicated before, the decrease in photosynthesis is usually caused by stomatal limitation under mild to moderate drought condition when both *G*_s_ and *C*_i_ decline while non-stomatal limitation is the main reason for the decrease in photosynthesis when *C*_i_ increases and *G*_s_ reaches a minimum inflection point (Pérez-López et al., 2012; Zhou et al., 2013). In our study, when *ψ*_w_ was above −1.5 Mpa, accompanied with a decrease of *G*_s_ and *E*, the *C*_i_ also significantly decreased under moderate drought stress treatments for 5 days, demonstrating that stomatal limitation primarily led to decrease of *P*_N_ in this period. As the degree of drought stress aggravated, when *ψ*_w_ was below −1.5, *P*_N_ kept linearly decreasing while *C*_i_ increased and *G*_s_ remained stable at approximately 70 mmol H_2_O m^−2^ s^−1^. *C*_i_ even increased when *P*_N_ continually went up linearly and a ‘V’ model is presented to describe the correlation between *C*_i_ and *ψ*_w_ (Fig. 3D). The changes of gas exchange parameters in this period implied the drop in photosynthesis activity might be caused by non-stomatal rather than stomatal limitation.

In order to further explore the relationship between drought stress and gas exchange parameters and photosynthetic activity, the rehydration treatment was conducted. The results showed that photosynthetic capacity impaired by drought stress can recover with different degrees after rehydration treatment. For instance, *ψ*_w_ and *P*_N_ in slight and moderate treatments recovered almost to the control level, while *G*_s_ or *E* only had a slight increase and *C*_i_ decreased significantly after 1 day of rehydration. The reversibility was dependent on not only the duration time of rehydration but also the degree and duration time of drought (Gomes et al., 2012; Šircelj et al., 2007). After 1 day of rehydration, *ψ*_w_ and *P*_N_ of apple leaves with severe stress treatment was still lower than that in slight and moderate treatments (Figs 2 and 3). However, the gap diminished significantly after 14 days' rehydration.

Drought stress significantly reduced CO_2_ assimilation rates at high *C*_i_, while only with a certain degree of lowering *P*_N_ rates at low *C*_i_. According to the model of photosynthesis (Farquhar and Sharkey, 1982; Sharkey et al., 2007), these results suggest that drought stress had a major impact on *J*_max_, with less effect on *V*_cmax_.

F_v_/F_m_, known as maximum quantum yield for primary photochemistry, could provide a simple and rapid way to evaluate when plants were exposed to stress environment (Henriques, 2009; Zai et al., 2012). Our study found F_v_/F_m_ in all three treatments decreased significantly compared with control after 33 days' drought treatment (Table 1). After rehydration for 14 days, F_v_/F_m_ of apple leaves under LS and MS stress can recover almost to control level while F_v_/F_m_ under SS stress did not recover and was still significantly lower than in control (Table 1). In addition, Φ_PSII_ decreased substantially, as well as *q*_P_ and *q*_L_, showing the capability of photochemistry conversion and linear electron flux were both sensitive to the degree and duration time of drought stress. Beyond these parameters, the rise in NPQ and the decline in F_m_ suggested the increase in energy dissipation through the xanthophyll cycle, which is the protecting mechanism to maintain normal photosynthesis in plants (Demmig-Adams and Iii, 1996; Jahns and Holzwarth, 2012). Interestingly, although NPQ in LS and MS went up, NPQ in the severe drought stress dropped dramatically. As has been reported previously, the increase in *Y*_(NPQ)_ expresses the attempt to dissipate excess energy while the increase in quantum yield of non-regulated heat dissipation and fluorescence emission [*Y*_(NO)_] signifies that excess energy fluxes are out of control and might produce photodamage to plants (Kramer et al., 2004). In our study, under drought conditions, the increase in both *Y*_(NPQ)_ and *Y*_(NO)_ compared with control also demonstrated the excess energy exceeded the regulatory ability of apple leaves and could not be effectively dissipated especially under SS. It might be a sign of irreversible cell dehydration and metabolism impairment (Kramer et al., 2004).

In the chlorophyll *a* fluorescence transient, the momentary maximum fluorescence intensity represents the subsequent kinetic bottlenecks of the electron transport chain (Strasser et al., 2010; Lazár et al., 2006). Schansker et al. (2005) reported that these limitations are the exchange of a reduced plastoquinone molecule with an oxidized one at the Q_B_ site (J-step) and the reoxidation of plastoquinol (PQH_2_, I-step). According to previous research on the OJIP-test, the change of chlorophyll fluorescence intensity in O-J, O-I, J-I phase can represent photosynthetic electron transport capacity between Q_A_ and Q_B_, Q_A_ and photosystem I (PSI) and Q_B_ and PSI acceptors, respectively (Stirbet and Govindjee, 2011). In the present study, the relationship was studied between the electron transport capacity and *ψ*_w_, the results implied the action side of drought stress was mainly on the electron transport from Q_A_ to Q_B_ for a higher correlation coefficient than that in the electron transport from Q_A_ to PSI and Q_B_ to PSI.

So what does it change inside photosynthetic apparatus and how does it lead to the decrease of electron transport capacity between Q_A_ to Q_B_? D1 protein has been proved to undertake biological function transferring photosynthetic electron from Q_A_ to Q_B_ (Roffey et al., 1994). In our study, D1 protein content decreased with the degree of water stress aggravated and the duration of water stress prolonged. After rehydration, D1 protein content recovered to some extent (Fig. 5). Previous research has shown that, to prevent the accumulation of photodamaged D1 and PSII, plants developed a repair process consisting of several steps as follows: proteolytic degradation of the D1 protein; synthesis of the precursor to the D1 protein (pre-D1); insertion of the newly synthesized precursor into the thylakoid membrane concomitant with the assembly of other PSII proteins; maturation of the D1 protein by C-terminal processing of pre-D1; and finally, assembly of the oxygen-evolving machinery (Aro et al., 1993, 2005). Under normal conditions, D1 protein content remains at a certain level by the balance between the damage and repair of D1 (Baena-González and Aro, 2002). Environmental stresses like salt stress and high temperature negatively influence the D1 content in PSII through inhibiting the repair as well as accelerating the damage. ROS was reported to be involved in the inhibition of repair (Takahashi and Murata, 2008). ROS generated by abiotic stresses blocks PSII repair by suppressing the transcription and translation of *psbA* genes encoding D1 (Nishiyama et al., 2001, 2004; Suleyman and Allakhverdiev, 2002).

Due to suppression of ROS on the transcription of *psbA* gene and translation D1 protein, the concentrations of H_2_O_2_ via O_2_^•^−^^ were probed in order to confirm if more ROS was induced by water stress. Apple leaves accumulated more ROS with different water stress treatments for 33 days than in control (Table 2). Since fixation of CO_2_ in the Calvin cycle is sensitive to environmental stress (Murata et al., 2007), it is likely to result in the limitation of photosynthesis and apple leaves absorbing more light energy than can be consumed through photosynthetic carbon fixation. The limitation of the photosynthetic fixation of CO_2_ decreases the utilization of NADPH, with a resultant decline in the level of NADP^+^ (Murata and Takahashi, 2008). Given that NADP^+^ is a major acceptor of electrons in PSI, depletion of NADP^+^ accelerates the transport of electrons from PSI to molecular oxygen with generation of H_2_O_2_ via O_2_^•^−^^ (Asada, 1999). Although plants have some protecting mechanisms that can dissipate excess energy such as non-photochemical quenching (Pieters and Tezara, 2003; Nabe et al., 2007), photorespiration (Cornic and Fresneau, 2002) and the Mehler reaction (Asada, 1999), the amount of energy dissipated by these mechanisms is still limited. When the degree and duration of water stress exceed the tolerance of plants, excess energy will lead to an increase in the production of ROS including O_2_^•^−^^ and H_2_O_2_.

During evolution, a series of antioxidant enzymes were developed to scavenge ROS induced by adverse environments. For instance, SOD plays a central role in the enzymatic defense system in removing O_2_^•^−^^ (Bowler et al., 1992) and CAT is indispensable in ROS-detoxification for its potential to directly dismutate H_2_O_2_ into H_2_O and O_2_^•^−^^ under stressed conditions (Garg and Manchanda, 2009). H_2_O_2_ is converted to water and oxygen via the ascorbate (AsA)-glutathione cycle and antioxidative enzymes (Blokhina et al., 2003). The ascorbate-glutathione cycle involves APX, which uses AsA as an electron donor to scavenge H_2_O_2_, so APX is also a key enzyme. Our results showed that antioxidant enzymes including CAT, SOD and POD activity increased as the degree of water stress aggravated and the duration of water stress prolonged this, but APX activity decreased under SS (Fig. 6). These results suggest that the AsA-glutathione cycle may not have a main role in clearing H_2_O_2_ in severe drought condition. Despite the fact that there were three antioxidant enzymes with higher activity, apple leaves under stress still accumulated more ROS than in control. This response to a water deficient environment indicates an insufficient protective mechanism in apple plants to clear excess ROS under stress for a long time. Consequently, the excess accumulation of ROS does harm to plant proteins, lipids, carbohydrates, DNA and ultimately results in irreversible damage and cell death (Apel and Hirt, 2004; Gill and Tuteja, 2010).

## CONCLUSION

Water deficiency in arid and semi-arid regions in northwestern China severely influences apple production. It is urgent to investigate how drought impacts the yield of apples and find a new understanding regarding this. As one of the most important biochemical reactions and the foundation of apple yield, photosynthesis decreases dramatically in drought environment.

After analysis of indicators and exploring their relationships among each other, it is concluded that photosynthetic activities are closely related to *ψ*_w_ and the response of photosynthetic apparatus to drought stress can be separated to two stages, and *ψ*_w_ with −1.5 MPa is the point to split the two stages.

In the first stage, the decline of photosynthetic CO_2_ assimilation under low drought stress was due to stomatal limitation, nevertheless, *V*_cmax_ decreased slightly. Together with stomatal close, the consumption of NADPH and *J*_max_ declined and caused a series of biochemical changes including overproduction of ROS, inhibition of D1 protein repair and eventual impairment of the electron transport chain.

In the second stage, the decline of photosynthetic CO_2_ assimilation under SS was due to non-stomatal limitation. After drought induced stomatal closure and inhibited CO_2_ assimilation, it then caused further PSII photoinhibition, dependent on the turnover of D1 protein, and over-reduced the electron transport chain, which increased the production of ROS (H_2_O_2_ and O_2_^•^−^^). The over-accumulated ROS inhibited the turnover of D1 protein and reduced electron Q_A_ to Q_B_. NADP^+^ and end electron acceptors may also both decline and in turn limit the synthesis of adenosine triphosphate (ATP) and the regeneration of RuBP (Lawlor and Tezara, 2009; Lin et al., 2009; Campos, et al., 2014). Thus, to interrupt Q_A_ to Q_B_, ATP shortage and low regeneration of RuBP we should impair the electron transport chain and the main non-stomatal factors under SS.

## MATERIALS AND METHODS

### Plant materials and drought stress treatments

The experiments were conducted in Northwest A&F University (NWAFU), Yangling, Shaanxi, China, located at 34°17′N, 108°04′E. Annual highest temperature was 36°C while the lowest was −11°C. The potted substrate was composed of soil mixture and organic matter (2:1, v/v; pH 7.5) with slow release organic-mineral fertilizer in growing season. The soil was collected from the top layer to 20 cm. The field capacity (FC) of potted substrate was 44.5%.

Three-year-old apple (*Malus domestica* Borkn. cv. Red Fuji) trees on M26 rootstocks were grown in plastic pots (245 mm diameter and 280 mm high). All the potted young trees were normally irrigated for 24 weeks under field conditions before drought stress was imposed. A plastic greenhouse (20 m×8 m×4 m) was utilized as the shelter to protect apple trees from the rain. The soil relative water content in control group was approximately 80% of maximal FC. Apple trees with LS, MS and SS were installed at 80%, 60% and 40% of the soil relative water content in control group. Four groups were arranged in a completely randomized design with eight replications.

### *ψ*_w_ measurements

For each treatment, six sunlight-exposed mature leaves were used. Referring to previous studies (Gomes-Laranjo et al., 2006; Jones, 2007; Šircelj et al., 2005), the *ψ*_w_ was measured with a pressure-bomb (Model 3000, Corp Santa Barbara, USA) between 8:00 h and 9:00 h.

### Gas exchange measurements

A portable photosynthesis system (LI-6400T, Li-Cor Inc., USA) with a 6400-02B light source (blue and red diode) was used to measure the photosynthetic gas exchange parameters including *P*_N_, *C*_i_, *E* and *G*_s_ *in vivo* on sunny days between 8:00 h and 9:00 h. Measurements were made under an artificial irradiance of 1000 μmol (photons) m^–2 ^s^–1^ at a temperature of 25°C using the fifth completely expanded leaf from the top of each plant. CO_2_ concentration and ambient water vapor pressure were kept at 350 μmol mol^−1^ and 1.30±0.15 kPa, respectively. To produce the *P*_N_/*C*_i_ curve, the CO_2_ concentration was set at 380 (for ambient leaves), 250, 200, 150, 100, 50, 350, 450, 550, 650 and 750 µmol mol^–1^ in turn, and the PPFD was kept at 1200 µmol (photons) m^–2^ s^–1^. The apparent carboxylation efficiency of Rubisco was estimated as the slope of the initial linear portion of each *P*_N_/*C*_i_ curve (Farquhar and Sharkey, 1982). *V*_cmax_ and *J*_max_ were calculated according to Sharkey et al. (2007). When *P*_N_ is Rubisco-limited, the response of *P*_N_ to *C*_i_ can be described using the following equation:

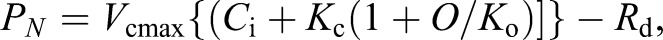
where *V*_cmax_ is the maximum velocity of Rubisco for carboxylation, *C*_i_ is the intercellular CO_2_ concentration,  Γ* is carbon dioxide compensation point, *K*_C_ is the Michaelis constant of Rubisco for carbon dioxide, O is the partial pressure of oxygen at Rubisco and *K*_O_ is the inhibition constant (usually taken to be the Michaelis constant) of Rubisco for oxygen, *R*_d_ is respiration rate. When *P*_N_ is limited by RuBP regeneration,

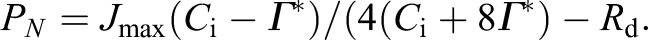
Based on the number of electrons required for NADP^+^ reduction, the conservative values of 4 and 8 are used here. Leaf temperature was 25±1°C by the temperature control system of leaf chamber.

### Chlorophyll fluorescence measurements

The same leaf was used for chlorophyll *a* fluorescence measurements right after gas exchange measurements. And measurements were conducted *in vivo* on sunny days (9:30 h to 11:30 h), with pulse amplitude modulation fluorometer (PAM-2500, Walz, Effeltrich*,* Germany).

#### Slow phase chlorophyll fluorescence transients (PSMT)

After a dark-adapted period (20 min) with dark leaf clip (DLC-8), the minimum fluorescence (F_0_) and maximum fluorescence (F_m_) were determined respectively using measure light [<1 µmol(photons) m^−2^ s^−1^] and a 0.8 s saturating pulse at 6000 µmol (photons) m^−2^ s^−1^. Actinic light of 619 μmol (photons) m^−2^ s^−1^ drives photosynthesis and gives F. After about 5 min, the steady state value of fluorescence (F_s_) was thereafter recorded and a second saturating pulse at 6000 µmol (photons) m^−2^ s^−1^ was imposed to determine F_m_ in the light adapted state (F_m_′). F_0_′ was basal fluorescence after 5 μmol (photons) m^−2^ s^−1^ of far-red irradiation at 720-730 nm for 4 s, which excites PSI and oxidizes the plastoquinone and Q_A_ pools associated with PSII. Also, F_v_/F_m_, actual photochemical efficiency (F_v_′/F_m_′), Φ_PSII_, *q*_P_, *q*_L_, NPQ, Y_(NPQ)_ and Y_(NO)_ were obtained from the measured report.

Chlorophyll *a* fluorescence transient (OJIP-test) was induced by a red light with a saturating light pulse of 3000 μmol (photons) m^−2^ s^−1^ using light-emitting diodes (LEDs), and fluorescence values were recorded for 350 ms with a time resolution of 10 μs. All of the leaves were dark-adapted for 20 min before measuring. The fluorescence intensity at 20 μs (considered as F_0_), 2 ms (F_J_) and 30 ms (F_I_) are intermediate levels, and maximum fluorescence or F_m_ (approximately 200 ms) was collected and used to calculate the parameters from JIP-test (Ceppi et al., 2011; Redillas et al., 2011).

### Determination of ROS

The H_2_O_2_ content and O_2_^•^−^^ generation rate were determined as described by Bai et al. (2010). Frozen tissues were homogenized in acetone at a ratio of 1.0 g sample to 2 ml ice-cold acetone. Titanium reagent (2% TiSO_4_) was added to a known volume of extract supernatant to give a Ti concentration of 2%. The Ti-H_2_O_2_ complex, together with unreacted Ti, was then precipitated by adding 0.2 ml of 17 M ammonia solution for every 1.0 ml of extract. The precipitate was washed five times with ice-cold acetone by resuspension, then drained and dissolved in 3 ml of 2.0 M H_2_SO_4_. Absorbance of the solution was measured at 410 nm against blanks that had been prepared similarly but without including plant tissue.

For evaluating the generation rate of O_2_^•^−^^, 1.0 g tissue was ground with 4.0 ml 65.0 mM phosphate buffer solution (PBS; pH 7.8) and centrifuged at 5000 ***g*** for 10 min. Afterward, 1.0 ml of supernatant was mixed with 0.9 ml 65 mM PBS (pH 7.8) and 0.1 ml 10.0 mM hydroxylamine hydrochloride, then incubated at 25°C for 20 min. Afterward, 17.0 mM sulfanilamide and 7 mM α-naphthylamine were added to the above mixture, which was then incubated at 25°C for 20 min. Light absorbance was measured at 530 nm. A standard curve with the nitrogen dioxide radical (NO_2_^−^) was used to calculate the production rate of O_2_^•^−^^.

### Extraction and assay of activities by CAT, SOD, POD and APX

Fresh tissue samples (0.1 g each) were homogenized with 5% (w/v) polyvinylpyrrolidone and homogenized with 1.8 ml of 100 mM potassium phosphate buffer (pH 7.0) containing 1.0 mM EDTA and 0.3% Triton X-100. The homogenates were centrifuged at 13,000 ***g*** for 20 min at 4°C and the supernatants were used for enzyme assays.

CAT activity was determined by monitoring the decrease in absorbance at 240 nm due to decomposition of H_2_O_2_ (Chance and Maehly, 1955). The 1.0 ml reaction mixture contained 39 mM potassium phosphate buffer (pH 7.0), 10 mM H_2_O_2_ and 20.0 μl of enzyme extract. This reaction was initiated by adding H_2_O_2_.

SOD activity was assayed by monitoring the inhibition of the photochemical reduction of nitro blue tetrazolium (NBT) according to the methods of Dhindsa et al. (1981). The 1.0 ml reaction mixture contained 50.0 mM potassium phosphate buffer (pH 7.8), 6.5 mM methionine, 50.0 μM NBT, 10.0 μM EDTA, 20.0 μM riboflavin, and 20.0 μl of enzyme extract. A reaction mixture lacking enzyme served as the control. All mixtures were stirred under darkness in small glass test tubes, and then irradiated for 5 min by fluorescent lamps [160 μmol (photons) m^−2^ s^−1^]. After the reaction mixture turned from yellow to blue-black, its absorbance was measured at 560 nm. The mixture that lacked enzyme and had not been irradiated was used to zero the absorbance at 560 nm. One unit of SOD was defined as the amount of enzyme that produced 50% inhibition of NBT reduction under assay conditions.

POD activity was determined by monitoring the increase in absorbance at 470 nm based on oxidation reaction of guaiacol. The 1.0 ml reaction mixture contained 39.75 mM potassium phosphate buffer (pH 7.0), 10.0 mM H_2_O_2_, 10.0 mM guaiacol and 5 μl enzyme extract.

APX activity was measured by monitoring the decrease in absorbance at 290 nm. The mixture of 5 ml contained 50 mM Hepes-KOH (pH 7.6), 0.1 mM EDTA, 0.2 mM H_2_O_2_, 0.5 mM reduced AsA and enzyme extract. The reaction was initiated by adding H_2_O_2_. One unit of activity was the amount of APX that catalyzed the oxidation of 1 mmol ascorbate per min.

### Western blot analysis

Total protein extracts were obtained by grinding 100.0 mg of leaf tissue in 3.0 ml of protein extraction buffer [0.5 M Tris-HCl, pH 6.8, 5 M urea, 8% (w/v) SDS, and 20% β-mercaptoethanol]. Samples were centrifuged at 13,000 ***g*** for 10 min, and the supernatant was subjected to SDS-PAGE. For detection of the D1 protein, the samples were separated on a 15% polyacrylamide gel in Tris-Gly buffer and electroblotted onto a nitrocellulose membrane. Blots were reacted with a commercially available antibody generated against D1 protein (Agrisera, Vännäs, Sweden), diluted 1:5000, and an anti-chicken horseradish peroxidase-conjugated secondary antibody, diluted 1:5000.

### Statistical analysis

The data obtained from measurements of selected photosynthetic parameters of plant leaves were statistically processed with Microsoft Excel 2007. Differences were evaluated by one-way ANOVA with the Statistical Program for Social Science 19 (SPSS, Chicago, USA). Only ANOVA Tukey results are presented in the paper. Graphs were plotted with Origin pro 7.5.
